# Identification of serotype O3b and high-risk clone ST37 of *Klebsiella pneumoniae* revealed by comparative genomic analysis

**DOI:** 10.3389/fcimb.2024.1517125

**Published:** 2025-01-20

**Authors:** Sivaraman Gopalan Krishnan, Sudha Sajeev, Visnuvinayagam Sivam, Raja Swaminathan T., Rakshit Ojha, Bibek Ranjan Shome, Mark Holmes, Thanigaivel Sundaram, Ramesh M. D., Saranya Vinayagam, Suseela Meesala, Tabarak Malik, Pavan Kumar Dara

**Affiliations:** ^1^ Microbiology Fermentation and Biotechnology Division, Indian Council of Agricultural Research (ICAR)-Central Institute of Fisheries Technology, Kochi, Kerala, India; ^2^ Department of Disease Investigation, Indian Council of Agricultural Research (ICAR)-National Institute of Veterinary Epidemiology and Disease Informatics, Bangalore, Karnataka, India; ^3^ Department of Veterinary Medicine, University of Cambridge, Cambridge, United Kingdom; ^4^ Department of Biotechnology, Faculty of Science and Humanities, SRM Institute of Science and Technology (SRMIST), Tamil Nadu, Kattankulathur, India; ^5^ Instituto de Alta Investigación, Universidad de Tarapacá, Arica, Chile; ^6^ Department of Biotechnology, Saveetha School of Engineering, Saveetha Institute of Medical and Technical Sciences, Chennai, India; ^7^ Department of Zoology, Vikrama Simhapuri University, Kavali, Andhra Pradesh, India; ^8^ Department of Biomedical Sciences, Jimma University, Jimma, Ethiopia; ^9^ Division of Research & Development, Lovely Professional University, Phagwara, Punjab, India

**Keywords:** *K. pneumoniae*, antibiotic resistant genes, whole genome sequencing, serotype, high-risk clone

## Abstract

**Background:**

Epidemiological risk factors such as the demography of a place, environment, food, livestock, and companion animals are known sources of *Klebsiella pneumoniae* infection. Whole-genome sequencing (WGS) has become a powerful tool to complement traditional microbiological characterization of foodborne pathogens. Moreover, *K. pneumoniae* has several species complexes (KpSC) and is very difficult to differentiate using routine microbiological methods. The present study aims to investigate the prevalence of *K. pneumoniae* in fish available in the retail market using WGS.

**Methods:**

Isolation of *K. pneumoniae*, identification of *K. pneumoniae* isolates, and determination of the minimum inhibitory concentration (MIC) were performed. Whole-genome sequencing of *K. pneumoniae* genomes and phylogenomic analysis were conducted for visual comparison of the genomes. Furthermore, genomes of non-human origin that were submitted from India to the NCBI database were downloaded and included in the comparative analysis.

**Results:**

The findings showed that many antibiotic-resistant genes (ARGs) are prominent, including *acrD, BaeR, cpxA, mdtB, mdtC, CRP, H-NS, KpnE, KpnF, KpnG, KpnH, acrA, acrB, marA, ramB, oqxA, oqxB, LptD*, and *emrR*. Four fish-sourced isolates had different blaSHV resistance gene variants. The presence of ARGs for aminoglycosides [aac(3)-IId], fluoroquinolones (oqxA, oqxB), and fosfomycin (fosA5, fosA6) in these *K. pneumoniae* isolates from fish sources was found. One of the CIFT-K6 isolates had the uncommon serotype of *K. pneumoniae* O3b with the high-risk clone “ST37.” The ST515 sequence type was present in two *K. pneumoniae* isolates (CIFT-K7 and CIFT-K8), but the O3b serotype and ST192 allele type were present in the CIFT-K10 isolate.

**Conclusion:**

To the best of our knowledge, this research study represents the first Indian report of *K. pneumoniae* linked to fish, specifically the high-risk clone ‘ST37’ and two other STs, 515 and 192. The most common plasmid type detected in all four isolates was IncFIB, and 75% of the isolates were IncFII and IncHI1B. The prevalence of ARGs linked to efflux pump resistance mechanisms is highlighted by the analysis of genome sequence data.

## 1 Introduction

*K. pneumoniae* is an important opportunistic pathogen that causes a wide range of infections in both community and healthcare settings (Keynan and Rubinstein, 2007). The World Health Organization has identified *K. pneumoniae* as an important threat to humanity because it can acquire and express resistance to various antimicrobial classes. This species is also listed as one of the pathogens in the *Enterococcus faecium, Staphylococcus aureus, Klebsiella pneumoniae, Acinetobacter baumannii, Pseudomonas aeruginosa, and Enterobacter species* (ESKAPE) group. It is a major cause of neonatal infection, urinary tract infection, septicemia, and soft tissue infection in hospitals. Community-acquired infections such as pyogenic liver abscesses, pneumonia, and meningitis are also serious health concerns caused by *K. pneumoniae* (Holt et al., 2015). Although humans are considered major reservoirs of these organisms, especially in the gastrointestinal tract, *Klebsiella* species have been identified in different habitats, including surface waters, sewage, industrial effluents, soil, and vegetation (Bagley, 1985). Moreover, they have also been associated with clinical mastitis in dairy cattle (Munoz et al., 2006), pneumonia in horses (Estell et al., 2016), and septicemia in pigs (Bidewell et al., 2018).

Despite the prevalence of *K. pneumoniae*, the role of foods in the prevalence and dissemination of this bacterium remains relatively underexplored. A prevalence of *K. pneumoniae* of approximately 21% was recently reported in raw meat and ready-to-eat food items from Singapore (Hartantyo et al., 2020). Several researchers have also documented food-borne outbreaks associated with *K. pneumoniae* (Calbo et al., 2011; Tambekar et al., 2011; Zhang et al., 2018). The selling of raw meat was cited as one of the transmission modes of virulence and especially antimicrobial resistance (AMR) genes from food to humans (Davis et al., 2015). In this context, exploring the molecular epidemiology and resistance development of isolates originating from food holds great significance in understanding the risk of *Klebsiella* infection and its transmission efficacy to humans (Wareth and Neubauer, 2021).

*K. pneumoniae* harbors a wide array of virulence factors, including capsules, fimbriae siderophores, lipopolysaccharides, aerobactin, salmochelin, yersiniabactin, colibactin, and various secretion systems (Lam et al., 2018). In addition, *K. pneumoniae* reportedly possesses multiple AMR genes, which severely limits therapeutic options for infections. The available data estimated that extended-spectrum beta-lactamase-producing and carbapenem-resistant strains of *K. pneumoniae* account for more than 90,000 infections and 7,000 deaths, respectively, in Europe alone (Wyres et al., 2020). With the increasing affordability and availability of next-generation sequencing (NGS), whole-genome sequencing (WGS) has emerged as a powerful tool to complement traditional microbiological characterization of foodborne pathogens. WGS provides the opportunity for in-depth analysis of organisms at the genomic scale by enabling rapid identification and detection of virulence traits and antimicrobial resistance by establishing phylogenetic relationships with related strains to infer epidemiology. Although many previous studies have examined the genomic features of *K. pneumoniae*, there is a paucity of data on the genomic features of *K. pneumoniae* of food origin. Therefore, in the present study, we reported the results of a comparative genomic analysis of *K. pneumoniae* isolates from fish to bridge the knowledge gaps in the genomic features of *K. pneumoniae* of food origin and to explore its virulence and antimicrobial resistance traits. Furthermore, the genomes of all available Indian *K. pneumoniae* isolates of food/environmental origin were compared for phylogenomic relatedness. To our understanding, this report constitutes the first such study from the Indian subcontinent.

## 2 Methods

### 2.1 Isolation of *Klebsiella pneumoniae*

A total of 37 different most commonly sold fish samples were collected from the major fish retail markets (n=10) in Cochin, Kerala, India in 2021. These fishes were caught in the Arabian Sea and its backwaters in Kochi, Kerala, India. The sample consisted of shrimp (10); pearlspot, *Ertoplus suratensis* (5); clam, *Villorita cyprinoides* (4); 3 each of catfish (*Tilapia mossambica*), sardine (*Sardinella longiceps*), and tilapia (*Tilapia mossambica*); 2 each of anchovy (*Stolephorus indicus*) and crab (*Portunus sanguinolentus*); 1 each of barracuda (*Pickhandle barracuda*), cod (*Epinephelus malabaricus*), milkfish (*Chanos chanos*), mullet (*Mugil cephalus*), sole (*Cyanoglossus macrostomus*), and scat (*Scatophagus argus*), which were collected and transported to the laboratory under cold conditions and processed within 4 hours of collection at the Microbiology, Fermentation and Biotechnology (MFB) Laboratory, Indian Council of Agricultural Research -National Institute of Veterinary Epidemiology and Disease Informatics (ICAR-CIFT), Cochin, Kerala. Gut and tissue samples from these fishes were aseptically removed, inoculated in Enterobacteriaceae enrichment (EE) broth in Mossel (BD Difco, Germany) liquid media for enrichment, and incubated at 37°C for 18-24 hrs. From this, a loopful of the inoculum was streaked onto the eosin methylene blue (EMB) agar medium (BD Difco, Germany). Pink mucoid colonies were selected and cultured on HiCrome™ Klebsiella Selective Agar Base (HiMedia, India). The media was chosen based on its ability to isolate and enumerate *Klebsiella* species chromogenically.

### 2.2 Determination of antimicrobial susceptibility

The identification of the *K. pneumoniae* isolates and determination of the minimum inhibitory concentration (MIC) was performed using a BD Phoenix™ M50 (BD Diagnostic Systems, Sparks, MD) and AST System (BD Diagnostic Systems, Sparks, MD). The gram-negative ID-AST combination NMIC/ID 55 panel was utilized with 19 different antibiotics, namely, amikacin (AN), amoxicillin/clavulanate (AMC), ampicillin (AM), aztreonam (ATM), cefazolin (CZ), cefepime (CPM), cefotaxime (CTX), cefoxitin (FOX), ceftazidime (CAZ), chloramphenicol (C), ciprofloxacin (CIP), gentamicin (GN), imipenem (IPM), levofloxacin (LVX), meropenem (MEM), piperacillin (PIP), piperacillin/tazobactam (PIP), tetracycline (TE), and trimethoprim/sulfamethoxazole (SXT), which represent 13 antimicrobial categories. The results were interpreted according to the method followed by Hsueh et al. (2010).

### 2.3 Whole-genome sequencing

The genomes of the isolates were extracted at the ICAR-NIVEDI using a MasterPure™ Gram Positive DNA Purification Kit (Lucigen, USA) following the manufacturer’s instructions. WGS was performed by using the Illumina HiSeq 2500 platform (Nasdaq, US). Paired ends were generated for all the isolates at the Sangers Institute, UK. In addition, all *K. pneumoniae* genomes of non-human origin that were submitted from India to the NCBI database were downloaded and included in the comparative analysis.

### 2.4 Assembly and annotation

Following a quality check of the WGS data with the FastQC tool (FastQC, A, 2015), the genomes were assembled using Shovill ver. 1.0.4 (https://github.com/tseemann/shovill), and a species check was performed using Kraken2 (https://ccb.jhu.edu/software/kraken/). The assembled genomes were annotated by Prokka v.1.14.5 (Seemann, 2014), and the annotation files were used for downstream analysis. To avoid annotation bias, all the *K. pneumoniae* genomes downloaded from the NCBI were reannotated with Prokka v1.14.5. In all the analyses, the genome of the *K. pneumoniae subsp. pneumoniae HS11286 (CP003200.1)* strain was included as a reference.

### 2.5 Detection of antimicrobial resistance, virulence factors, plasmid profiling and typing

AMR genes were identified via the Resistance Gene Identifier (RGI) (https://card.mcmaster.ca/analyze/rgi), while virulence genes were identified via the use of virulence factors of pathogenic bacteria (VFDB) (http://www.mgc.ac.cn/VFs/) from the genome. Additional virulence plasmid-associated K loci (capsule synthesis) and O antigen (lipopolysaccharide) serotype prediction, allele diversity, and multiple locus sequence type (MLST) of *K. pneumoniae* were screened using KleBorate (https://github.com/katholt/Kleborate). A search for plasmids in the genome was performed using the PlasmidFinder database available at https://bitbucket.org/genomicepidemiology/plasmidfinder_db/src/master/.

### 2.6 Phylogenomic analysis

For visual comparison of the genomes, a BLAST plot was generated with a Blast Ring Image Generator Alikhan et al. (2011) using *K. pneumoniae subsp. pneumoniae* HS11286 as the reference genome. Apart from the sequenced genomes, all the downloaded genomes were also included in the analysis. To construct a phylogenetic tree, all the *K. pneumoniae* genomes were aligned with MAFFT (Katoh et al., 2002). Poorly aligned regions were filtered out using Gblock v. 0.91 (Talavera and Castresana, 2007). Curated alignment was used as the input for IQ-TREE v.1.6.12 run along with ModelFinder, and a phylogenetic tree was drawn using the best-fit model GTR+F+R5 as per the Bayesian Information Criteria score. The tree was visualized using MEGA 7.0.26 as followed by Kumar et al. (2016).

### 2.7 Nucleotide sequence accession numbers/submission to the NCBI portal

The whole genome project has been submitted as a project with the accession ID PRJNA704514. The whole-genome shotgun project has been deposited in DDBJ/ENA/GenBank under the accession numbers JAGIZC000000000 (CIFT-K6), JAGIZD000000000 (CIFT-K7), JAGIZE000000000 (CIFT-K8), and JAGIZF000000000 (CIFT-K10).

## 3 Results

### 3.1 Phenotypes and antimicrobial susceptibility

The purple-magenta mucoid colonies cultured on HiCrome™ Klebsiella Selective Agar Base medium were considered to be *Klebsiella* sp. phenotypically. Non-duplicate isolates from each sample were identified as *K. pneumoniae* in a BD Phoenix™ M50. All the isolates were susceptible to the antibiotics tested, while resistance to ampicillin was inherent to *K. pneumoniae*. Interestingly, all the isolates had intermediate MICs for CZ, cefotaxime, CIP, and LVX. The MIC results for these isolates are presented in **Table 1**.

**Table 1:** Minimum inhibitory concentration (MIC) levels of antibiotics calculated using a BD Pheonix™ M50 as per CLSI (2021) for the *K.pnemoniae* isolates.

SL. No	Isolate ID	Code	Organism	Remarks	Amikacin	Clavulanate	Ampicillin	Aztreonam	Cefazolin	Cefepime	Cefoperazone/Sulbactam	Cefotaxime	Cefoxitin	Ceftazidime	Chloramphenicol	Ciprofloxacin	Colistin	Gentamicin	Imipenem	Levofloxacin	Meropenem	Piperacillin	Piperacillin/ Tazobactum	Tetracycline	Trimethoprim/ Sulfactum
					AN	AMC	AM	ATM	CZ	FEP	SCP	CTX	FOX	CAZ	C	CIP	CL	GM	IPM	LVX	MEM	PIP	TZP	TE	SXT
					8 to 32	4/2 to 16/8	4 to 16	2 to 16	4 to 16	2 to 16	0.5/8 to 16/8	4 to 32	4 to 16	1 to 16	4 to 16	0.5 to 2	0.5 to 2	2 to 8	1 to 8	1 to 4	1 to 8	4 to 64	4/4 to 64/4	2 to 8	0.5/9 to 2/3
1	Crab	CIFT_6	*K.pneumoniae*	Non-ESBL	≤8	≤4/2	>16	≤2	≤4	≤	≤0.5/8	≤4	≤4	≤1	≤4	≤0.5	≤0.5	≤2	≤1	≤1	≤1	8	≤4/4	≤2	≤0.5/9.5
					S	S	R	S	I	S	S	I	S	S	S	I	S	S	S	I	S	S	S	S	S
2	Mackerel	CIFT_7	*K.pneumoniae*	Non-ESBL	≤8	≤4/2	>16	≤2	≤4	≤	≤0.5/8	≤4	≤4	≤1	≤4	≤0.5	≤0.5	≤2	≤1	≤1	≤1	≤4	≤4/4	≤2	≤0.5/9.5
					S	S	R	S	I	S	S	I	S	S	S	I	S	S	S	I	S	S	S	S	S
3	Tilapia	CIFT_8	*K.pneumoniae*	Non-ESBL	≤8	≤4/2	>16	≤2	≤4	≤	≤0.5/8	≤4	≤4	≤1	≤4	≤0.5	≤0.5	≤2	≤1	≤1	≤1	≤4	≤4/4	≤2	≤0.5/9.5
					S	S	R	S	I	S	S	I	S	S	S	I	S	S	S	I	S	S	S	S	S
4	Clam	CIFT_10	*K.pneumoniae*	Non-ESBL	≤8	≤4/2	>16	≤2	≤4	≤	≤0.5/8	≤4	≤4	≤1	≤4	≤0.5	≤0.5	≤2	≤1	≤1	≤1	4	≤4/4	≤2	≤0.5/9.5
					S	S	R	S	I	S	S	I	S	S	S	I	S	S	S	I	S	S	S	S	S

### 3.2 Antimicrobial resistance genes in *K. pneumoniae*

The occurrence of resistance genes in the genomes of *K. pneumoniae* was analyzed using the CARD database. The results revealed the presence of 30 genes (**Table 2**). Genome analysis revealed no genes related to the cephalosporin-associated CTX-M group. Despite the absence of prominent ESBL genes, different allelic variants of beta-lactamase sulfhydryls (*bla*_SHV_) were observed. *bla*_SHV-1_ was present in CIFT-K7 and K8. *bla*_SHV-110_ was detected in CIFT-K6 and *bla*_SHV-60_ was detected in CIFT-K10. The aminoglycoside efflux pump gene *acrD* was detected in all the strains. The CIFT-K10 isolate harbored the plasmid-encoded aminoglycoside acetyltransferase gene *aac(3)-IId* along with the reference isolate Kp_HS11286, while *aac(6’)-Ib9* was detected in Kp_C13. The phosphotransferase genes *aph(3”)-Ib* and *aph(6)-Id* were present only in the reference genome. Even one of the 16S rRNA methylase genes (*rmtB*) was present in the isolate Kp_HS11286. The *baeR*, a response regulator that promotes the expression of *MdtABC*, was present in all four isolates. One of the porins linked with the outer membrane gene *OmpK37* was also detected in all the isolates. Among the other genomes that were used for comparison, *acrA, acrB, acrD*, *marA, ramA*, and *baeR* were present universally. Multidrug resistance antibiotic efflux genes (*KpnEF, LptD, msbA, mdtB, mdtC, CRP*, and *H-NS*) were common to all the non-human isolates.

**Table 2:** Antimicrobial resistance gene determination of the *K.pneumoniae* isolates from fish in comparison with the reference *K. pneumoniae* genome sequences from India.

Resistance Mechanism	Drug Class	AMR Gene	CIFTK6	CIFTK7	CIFTK8	CIFTK10	Kp_AWD5	Kp_C13	Kp_EGD-HP19-C	Kp_HPCN17	Kp_HPCN22	Kp_HPCN5	Kp_HS11286	Kp_KBG6.2	Kp_ME30	Kp_PL1-RCS238	Kp_PVN-1
Antibiotic inactivation	Aminoglycoside and aminoglycoside-modifying enzymes (ame)	AAC(3)-IId	–	–	–	**+**	–	–	–	–	–	–	**+**	–	–	–	–
		AAC(6’)-Ib9	–	–	–	–	–	**+**	–	–	–	–	–	–	–	–	–
		APH(3”)-Ib	–	–	–	–	–	–	–	–	–	–	**+**	–	–	–	–
		APH(6)-Id	–	–	–	–	–	–	–	–	–	–	**+**	–	–	–	–
		aadA2	–	–	–	–	–	**+**	–	–	–	–	**+**	–	–	–	–
Antibiotic efflux		acrD	**+**	**+**	**+**	**+**	**+**	**+**	**+**	**+**	**+**	**+**	**+**	**+**	**+**	**+**	**+**
Antibiotic inactivation		rmtB	–	–	–	–	–	–	–	–	–	–	**+**	–	–	–	–
Antibiotic efflux	Aminocoumarin; aminoglycoside	BaeR	**+**	**+**	**+**	**+**	–	**+**	**+**	**+**	**+**	**+**	**+**	**+**	**+**	**+**	**+**
		cpxA	**+**	**+**	**+**	**+**	**+**	**+**	**+**	**+**	–	**+**	**+**	**+**	**+**	**+**	–
Antibiotic efflux	Aminocoumarin	mdtB	**+**	**+**	**+**	**+**	–	**+**	**+**	**+**	**+**	**+**	**+**	**+**	**+**	**+**	**+**
		mdtC	**+**	**+**	**+**	**+**	–	**+**	**+**	**+**	**+**	**+**	**+**	**+**	**+**	**+**	**+**
Antibiotic inactivation	Peptide	ArnT	**+**	**+**	**+**	**+**	**+**	**+**	**+**	**+**	**+**	**+**	**+**	**+**	**+**	**+**	**+**
		eptB	**+**	**+**	**+**	**+**	**+**	**+**	**+**	**+**	**+**	**+**	**+**	**+**	**+**	**+**	**+**
Antibiotic efflux	Penam; macrolide; fluoroquinolone	CRP	**+**	**+**	**+**	**+**	**+**	**+**	**+**	**+**	**+**	**+**	**+**	**+**	**+**	**+**	**+**
Antibiotic inactivation	Macrolide	EreA2	–	–	–	–	–	–	**+**	–	–	–	–	–	–	–	–
		mphA	–	–	–	–	–	**+**	–	–	–	–	–	–	–	–	–
Antibiotic inactivation	Streptogramin; macrolide; lincosamide	ErmB	–	–	–	–	–	**+**	–	–	–	–	–	–	–	–	–
Antibiotic efflux	Fluoroquinolone; penam; macrolide; cephamycin; cephalosporin; tetracycline	H-NS	**+**	**+**	**+**	**+**	**+**	**+**	**+**	**+**	**+**	**+**	**+**	**+**	**+**	**+**	**+**
Antibiotic inactivation	Cephalosporin	CTX-M-14	–	–	–	–	–	–	–	–	–	–	**+**	–	–	–	–
		CTX-M-15	–	–	–	–	–	**+**	–	–	–	–	–	–	–	–	–
Antibiotic inactivation	Fosfomycin	fosA5	–	–	–	**+**	–	**+**	–	–	–	–	–	–	–	**+**	**+**
		fosA6	**+**	**+**	**+**	–	**+**	–	**+**	**+**	**+**	**+**	**+**	**+**	**+**	–	–
Antibiotic inactivation	Cephalosporin; penam; carbapenem; monobactam	KPC-1	–	–	–	–	–	–	–	–	–	–	**+**	–	–	–	–
Antibiotic efflux	Peptide; aminoglycoside; tetracycline; rifamycin; macrolide; cephalosporin	KpnE	**+**	**+**	**+**	**+**	**+**	**+**	**+**	**+**	**+**	**+**	**+**	**+**	**+**	**+**	**+**
		KpnF	**+**	**+**	**+**	**+**	**+**	**+**	**+**	**+**	**+**	**+**	**+**	**+**	**+**	**+**	**+**
Antibiotic efflux	Peptide; penam; aminoglycoside; macrolide; fluoroquinolone; carbapenem; cephalosporin; penem	KpnG	**+**	**+**	**+**	**+**	**+**	**+**	**+**	**+**	**+**	**+**	**+**	**+**	**+**	**+**	–
		KpnH	**+**	**+**	**+**	**+**	**+**	**+**	**+**	**+**	**+**	**+**	**+**	**+**	**+**	**+**	–
Antibiotic efflux	–	cmlA9	–	–	–	–	–	–	–	–	–	–	**+**	–	–	–	–
Reduced permeability to antibiotic	Cephamycin; penem; monobactam; penam; cephalosporin; carbapenem	OmpK37	**+**	**+**	**+**	**+**	–	**+**	**+**	**+**	**+**	**+**	**+**	**+**	**+**	**+**	**+**
Antibiotic efflux	Tetracycline; cephalosporin; glycylcycline; penam; phenicol; rifamycin; triclosan; fluoroquinolone	acrA	**+**	**+**	**+**	**+**	**+**	**+**	**+**	**+**	**+**	**+**	**+**	**+**	**+**	**+**	**+**
		acrB	**+**	**+**	**+**	**+**	**+**	**+**	**+**	**+**	**+**	**+**	**+**	**+**	**+**	**+**	**+**
Antibiotic efflux; reduced permeability to antibiotic	Tetracycline; cephalosporin; glycylcycline; penam; penem; carbapenem; phenicol; cephamycin; rifamycin; monobactam; triclosan; fluoroquinolone	marA	**+**	**+**	**+**	**+**	**+**	**+**	**+**	**+**	**+**	**+**	**+**	**+**	**+**	**+**	**+**
		ramA	**+**	**+**	**+**	**+**	**+**	**+**	**+**	**+**	**+**	**+**	**+**	**+**	**+**	**+**	**+**
Antibiotic efflux	Tetracycline; diaminopyrimidine; glycylcycline; nitrofuran; fluoroquinolone	oqxA	**+**	**+**	**+**	**+**	**+**	**+**	**+**	**+**	**+**	**+**	–	**+**	**+**	**+**	–
		oqxB	**+**	**+**	**+**	**+**	**+**	–	**+**	**+**	**+**	**+**	–	**+**	**+**	**+**	–
Antibiotic efflux	Carbapenem; peptide; aminocoumarin; rifamycin	LptD	**+**	**+**	**+**	**+**	**+**	**+**	**+**	**+**	**+**	**+**	**+**	**+**	**+**	**+**	**+**
Antibiotic efflux	Nitroimidazole	msbA	**+**	**+**	**+**	**+**	**+**	**+**	**+**	**+**	**+**	**+**	**+**	**+**	**+**	**+**	**+**
Antibiotic inactivation	Cephalosporin; carbapenem; penam	OXA-232	–	–	–	–	–	**+**	–	–	–	–	–	–	–	–	–
Reduced permeability to antibiotic	Penem; penam; cephamycin; cephalosporin; carbapenem; monobactam	OmpA	**+**	**+**	**+**	**+**	**+**	**+**	**+**	**+**	**+**	**+**	**+**	**+**	**+**	**+**	**+**
Antibiotic inactivation	Penam; carbapenem; cephalosporin	SHV-1	–	**+**	**+**	–	–	–	–	–	–	**+**	–	–	–	–	–
		SHV-106	–	–	–	–	–	**+**	–	–	–	–	–	–	–	–	–
		SHV-110	**+**	–	–	–	–	–	–	**+**	**+**	–	–	–	–	–	–
		SHV-120	–	–	–	–	–	–	–	–	–	–	–	–	–	**+**	–
		SHV-134	–	–	–	–	–	–	**+**	–	–	–	–	–	–	–	–
		SHV-182	–	–	–	–	–	–	–	–	–	–	**+**	–	–	–	–
		SHV-187	–	–	–	–	**+**	–	–	–	–	–	–	**+**	**+**	–	**+**
		SHV-60	–	–	–	**+**	–	–	–	–	–	–	–	–	–	–	–
Antibiotic inactivation	Penam; monobactam; penem; cephalosporin	TEM-181	–	–	–	–	–	**+**	–	–	–	–	**+**	–	–	–	–
Antibiotic inactivation	Rifamycin	arr-2	–	–	–	–	–	**+**	–	–	–	–	–	–	–	–	–
Antibiotic inactivation	Phenicol	catI	–	–	–	–	–	**+**	–	–	–	–	–	–	–	–	–
Antibiotic target replacement	Diaminopyrimidine	dfrA12	–	–	–	–	–	**+**	–	–	–	–	**+**	–	–	–	–
Antibiotic efflux	Fluoroquinolone	emrR	**+**	**+**	**+**	**+**	**+**	**+**	**+**	**+**	**+**	**+**	**+**	**+**	**+**	**+**	–
Antibiotic efflux	Acridine	qacEdelta1	–	–	–	–	–	**+**	–	–	–	–	**+**	–	–	–	–
Antibiotic target replacement	Sulfonamide	sul1	–	–	–	–	–	**+**	–	–	–	–	–	–	–	–	–
		sul2	–	–	–	–	–	–	–	–	–	–	**+**	–	–	–	–
Antibiotic efflux	Tetracycline	tet(G)	–	–	–	–	–	–	–	–	–	–	**+**	–	–	–	–

Colour shading indicates the presence of antibiotic resistance genes.

### 3.3 Virulence genes in the genomes of *K. pneumoniae*

Among those in the adherence class, the majority of the type 3 fimbriae virulence genes, *viz., mrkA*, *B, C, D, F, H, I*, and *J*, were present in the four genomes of CIFT-K6, K7, K8, and K10, while *mrkH* was absent in CIFT-K7 and K8. Operons linked to type I fimbriae-*fimA-I* and *K* were present in all four isolates. The *rmpA* gene, a transcriptional regulator gene associated with the mucoid phenotype capable of causing community-acquired infection, was absent in all the studied genomes and the reference genomes included in the study. The capsule virulence factor responsible for antiphagocytosis was present, while the capsule polysaccharide (Vibrio)-related virulence gene *cpsA* was absent. The efflux pump virulence factor *AcrAB* was found in 100% of the isolates. The virulence factors aerobactin, namely, *iucA, B, C* and *D*, were also absent from all four genomes, while *iutA* was present in all the *Klebsiella* isolates. The genes (*entB*, *entC*, *entD*, *entE*, *entF*, *entS*, *fepB*, *fepB*, *fepC*, *fepD*, *fepG* and *fes*) encoding the iron-uptake enterobactin siderophores were present in all the isolates. The details of the ORFs of the virulence genes analyzed using the VFDB database are given in **Supplementary Table S1**. Salmochelin virulence factors (*iroB*, *iroC*, and *iroD*), which are of the same iron uptake class, were absent, while the ORFs corresponding to the virulence genes *iroE* and *iroN* were present in all four genome sequences. Genes corresponding to allantoin metabolism, *allA*, *B, C, D, R* and *S*, were completely absent. Virulence genes (*fyuA*, *irp1*, *irp2*, *ybtA*, *ybtE*, *ybtP*, *ybtQ*, *ybtS*, *ybtT*, *ybtU*, and *ybtX*, which are yersiniabactin factors) were all absent in the studied isolates. The reference genomes Kp_HS11286 and Kp_C13 included genes corresponding to yersiniabactin, while Kp_C13 also included aerobactin. Among all the food/environmental genomes studied from India, none of them contained colibactin, salmochelin, or hypermucoidy virulence genes. The confidence intervals for the K locus in the isolates were above 98% in all four genomes studied: CIFT-K6, 99.71%; CIFT-K7, 98.95%; CIFT-K8, 98.95%; and CIFT-K10, 99.93%. The K locus identified for the CIFT-K7 and K8 isolates was KL8, while it was KL38 for CIFT-K6 and KL-111 for CIFT-K10. The O-locus confidence percentages were as follows: CIFT-K6, 99.28%; CIFT-K7, 98.51%; CIFT-K8, 98.51%; and CIFT-K10, 99.23%. Serotype analysis of the WGS data revealed three types of *wzi* alleles (196, 334, and 113) and two types of O loci, O3b and O1v2. The typing results for kleborate are presented in **Table 3**.

**Table 3:** Analysis of the diversity of alleles in *K.pneumoniae* isolates using Kleborate.

Strain	wzi	K serotype	K serotype identity	O serotype	O serotype identity
CIFT-K6	196	KL38	99.71%	O3b	99.28%
CIFT-K7	334	KL8	98.95%	O1v2	98.51%
CIFT-K8	334	KL8	98.95%	O1v2	98.51%
CIFT-K10	113	KL111	99.93%	O3b	99.23%

### 3.4 BLAST-ATLAS

**Figure 1:**
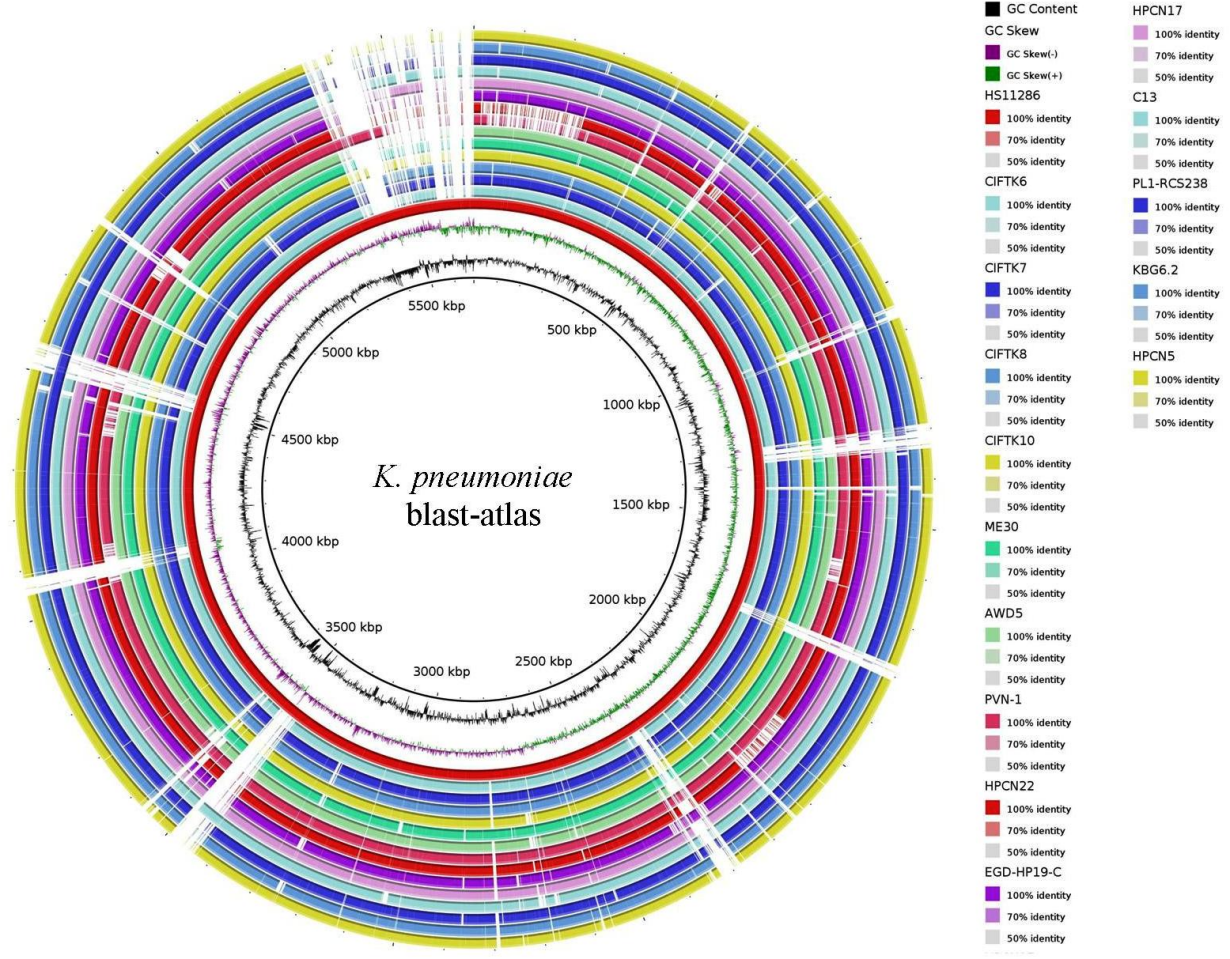
BLAST-ATLAS for the *K. pneumoniae* isolate genomes with a reference *K. pneumoniae* HS11286 strain.

For ease of comparative visual assessment of *K. pneumoniae*, a BLAST-ATLAS was generated for all the genomes, with *K. pneumoniae* HS11286 serving as a reference (**Figure 1**). Close inspection of the genomic map revealed multiple gapped regions in all the genomes (except for the reference), perhaps indicating the draft nature of the genomes. However, gapped regions between 3,000 and 3,600 kb, 4,500 and 4,600 kb, and 5,300 and 5,500 kb were also accompanied by perturbations in GC content, indicating possible horizontal gene transfer.

### 3.5 Whole-genome MLS typing

Multiple locus sequence typing (MLST) using the WGS data revealed that two of the four genomes belonged to ST515, that of the CTFT-K6 isolate was typed as ST37, and that of the fourth isolate, CIFT-K10, was of the ST192 type. The other *K. pneumoniae* genomes belonged to 10 different STs. The allelic profiles and sequence types of the strains in comparison with the reference genomes are presented in **Table 4**. Overall, we noticed wide diversity in the STs of the *K. pneumoniae* isolates studied. In the present study, ST37 was associated with *bla*_SHV-110_, ST515 with *bla*_SHV-1_, and *bla*_SHV-60_ with ST192. All these clones are linked with broad-spectrum beta-lactamases in *K. pneumoniae*. Studies have shown a close association between blaOXA-1 and ST37 *K. pneumoniae* isolates, as reported by Li et al. (2017).

**Table 4:** MLST analysis of the *K.pneumoniae* isolates from the retail market fish in comparison with other food/environmentally sourced *K. pneumoniae* genomes from India.

Isolate/Genome	Sequence type	Genes						
		*gapA*	*infB*	*mdh*	*pgi*	*phoE*	*rpoB*	*tonB*
CIFTK6	ST37	2	9	2	1	13	1	16
CIFTK7	ST515	2	1	1	1	1	1	4
CIFTK8	ST515	2	1	1	1	1	1	4
CIFTK10	ST192	4	1	1	2	46	4	4
Kp_AWD5	ST200	2	1	2	1	12	1	68
Kp_C13	ST231	2	6	1	3	26	1	77
Kp_EGD-HP19-C	ST1377	2	1	11	4	9	4	13
Kp_HPCN17	ST2701	4	1	1	1	1	1	363
Kp_HPCN22	ST2701-3LV	–	1	–	1	1	–	363
Kp_HPCN5	ST3689	2	3	1	1	2	1	14
Kp_HS11286	ST11	3	3	1	1	1	1	4
Kp_KBG6.2	ST22	2	3	1	1	1	4	4
Kp_ME30	ST1728	2	1	20	1	4	5	110
Kp_PL1-RCS238	ST557	4	5	1	1	9	1	25
Kp_PVN-1	ST1107-1LV	4	38	1	1	7	–	6

### 3.6 Phylogenomic analysis

**Figure 2:**
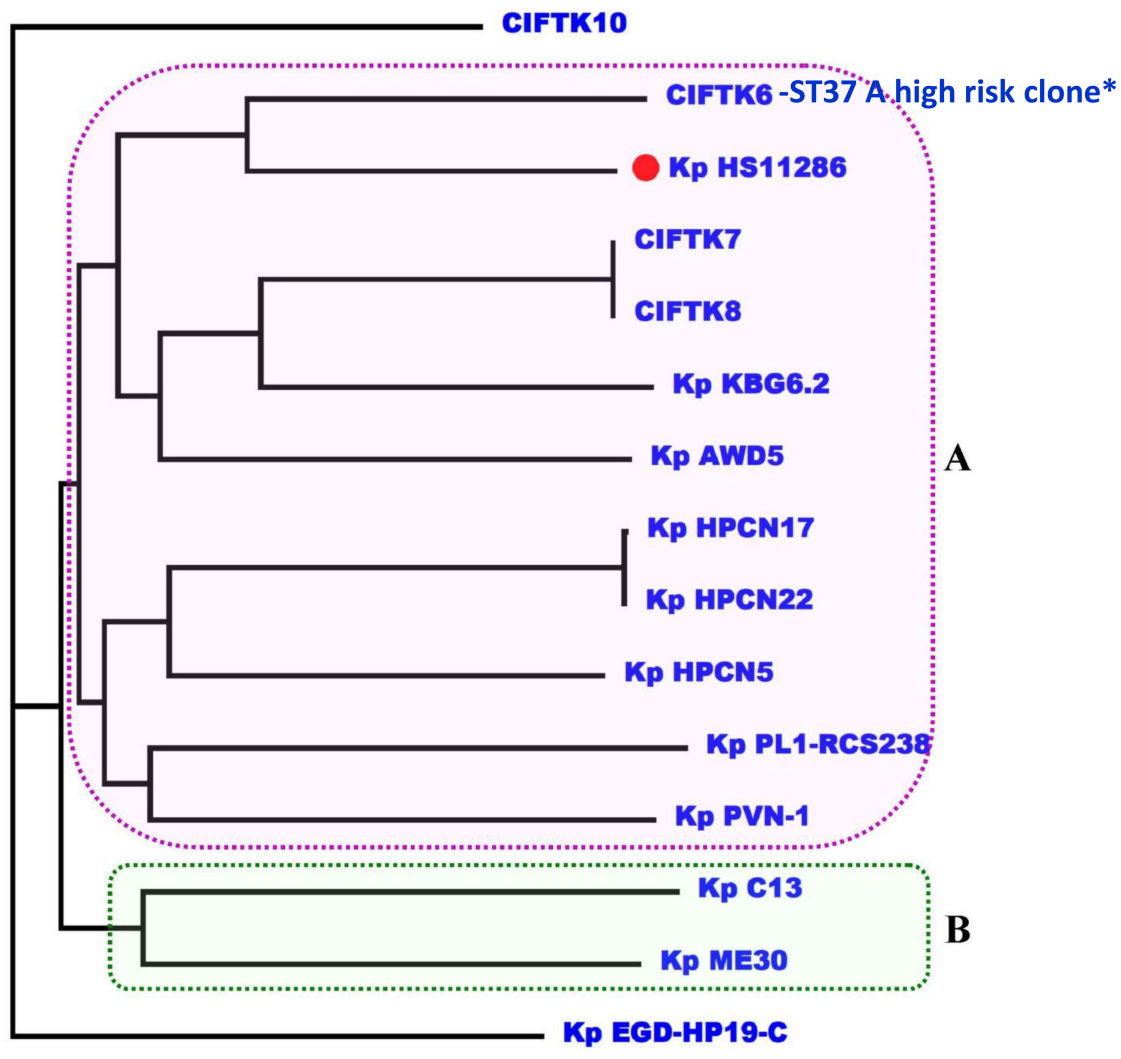
Phylogenomic analysis of the *K. pneumoniae* genomes using the GTR+F+R5 model showing that CIFT6-ST37 is a high-risk clone in cluster A. *indicates the high risk clone of K.pneumoniae ST37 in the phylogenetic cluster A.

To infer the phylogenetic relatedness among the genomes, we constructed a phylogenetic tree using the GTR+F+R5 model (**Figure 2**). The phylogenetic tree revealed that the isolates that we sequenced in this study (CIFT-K6, 7, 8, and 10) were positioned considerably farther from the other genomes of *K. pneumoniae* (excluding the reference strain) that were included in this study. Among the CIFT isolates, CIFT-K6 was closely related to the reference strain of *K. pneumoniae* HS11286. Broadly, there were two large clusters (‘A’ and ‘B’) and two outlier genomes (CIFT-K10 and *K. pneumoniae* EGD-HP-19-C). Cluster ‘A’ harbored the rest of the CIFT genomes (CIFT-K6, 7, and 8), while cluster ‘B’ harbored the other genomes that we included in this study. The results indicated that *K. pneumoniae* genomes of fish origin were distinct in lineage compared to other non-human *K. pneumoniae* genomes.

### 3.7 Plasmids in the genomes of *K. pneumoniae*

Bacterial whole-genome sequence analysis of the four isolates revealed the presence of various plasmid types. The sequenced *K. pneumoniae* isolates and reference genomes studied using PlasmidFinder with diverse replicons are summarized in **Table 5**. IncFIB was the most prominent plasmid type among the four isolates, followed by IncFII and IncHI1B in 75% of the isolates. The CIFT-K7 and CIFT-K8 isolates, which are of the ST515 type, contained the plasmid replicons Col(pHAD28), Col440II, and IncR. The CIFT-K10 isolate contained the IncFIB plasmid, while the CIFT-K6 isolate contained the Col440I, IncFIA(HI), and IncQ2 plasmids. Among the other food/environmental genomes compared, the most prominent replicon type was IncFII (pKP91). A maximum of seven hits to replicons in the database were found in the reference genome strain of *K. pneumoniae subsp. pneumoniae* HS11286, while Kp_ME30 had four hits, and Kp_PL1-RCS238 had two hits. One of three reference isolates (Kp_EGD-HP19-C, Kp_HPCN17, and Kp_PVN-1) presented one plasmid replicon each for pENTAS02, IncFIA(HI1), and IncFIB(pKPHS1), respectively.

**Table 5:** A comparison of putative plasmids from the fish samples with reference genomes of *K. pneumoniae* using PlasmidFinder.

Isolate	Replicon																				
	Num_Found (n)	Col(KPHS6)	Col(pHAD28)	Col156	Col440II	Col440I	ColKP3	IncC	IncFIA(HI1)	IncFIA	IncFIB(K)(pCAV1099-114)	IncFIB(K)	IncFIB(pKPHS1)	IncFIB(pNDM-Mar)	IncFIB(pQil)	IncFII(pAMA1167-NDM-5)	IncFII(pKP91)	IncHI1B(pNDM-MAR)	IncQ2	IncR	pENTAS02
CIFT-K6	6	–	–	–	–	100	–	–	100	–	100	–	–	–	–	–	100	100	98.89	–	–
CIFT-K7	6	–	100	–	99.29	–	–	–	–	–	100	–	–	–	–	–	100	100	–	100	–
CIFT-K8	6	–	100	–	99.29	–	–	–	–	–	100	–	–	–	–	–	100	100	–	100	–
CIFT-K10	1	–	–	–	–	–	–	–	–	–	100	–	–	–	–	–	–	–	–	–	–
Kp_C13	6	–	100	–	–	–	100	–	–	100	–	–	–	–	100	100	100	–	–	–	–
Kp_EGD-HP19-C	1	–	–	–	–	–	–	–	–	–	–	–	–	–	–	–	–	–	–	–	98.67
Kp_HPCN17	1	–	–	–	–	–	–	–	100	–	–	–	–	–	–	–	–	–	–	–	–
Kp_HS11286	7	100	100	100	–	–	–	100	–	–	–	–	100	–	–	–	100	–	–	100	–
Kp_ME30	4	–	–	–	–	100	–	–	–	–	100	–	–	100	–	–	–	100	–	–	–
Kp_PL1-RCS238	2	–	–	–	–	–	–	–	–	–	–	100	–	–	–	–	100	–	–	–	–
Kp_PVN-1	1	–	–	–	–	–	–	–	–	–	–	–	100	–	–	–	–	–	–	–	–

## 4 Discussion

Kerala is one of the leading fish-eating states, with almost 85% of the population consuming a variety of freshwater and seawater fishes. The local retail fish markets in Cochin are influenced by the tastes and preferences of consumers (Shyam, 2020). Small and medium fishes are the mainstays of daily cuisine. Varieties such as sardines, mackerel, tilapia, shrimp, crab, clam, mullet, and ertoplus are the most common fishes sold on the market. As fish are among the prominent foods in Kerala cuisine, tracing, identifying, and characterizing the biological contaminants that may affect human health are highly important.

*Klebsiella* species are ubiquitous in the natural environment and are widely distributed in the gastrointestinal tracts of humans and animals (Gundogan, 2014). It is an important opportunistic pathogen of humans and has acquired international heterogeneity due to the increasing occurrence of AMR. In general, three distinct species of *Klebsiella* are classified into different phylogroups: *K. pneumoniae* as KpI, *Klebsiella quasipneumoniae* as KpII, and *Klebsiella varicola* as KpIII (Dworkin et al., 2006). *K. pneumoniae* is associated with a wide range of hosts, from humans to livestock, and different environmental niches (Brisse et al., 2014). In the present study, we reported the genomic features of *K. pneumoniae* of fish origin and evaluated them in comparison with other food/environmentally sourced genomes of *K. pneumoniae* available in public databases from the Indian subcontinent for the first time.

A total of 6 K*.pneumoniae*, 15 *E.coli*, 3 *Pseudomonas aeruginosa*, 2 *Acinetobacter baumanni/calcoacetius* complex, 1 *Pseudomonas putida*, 1 *Aeromonas veronii bv veronii*, and 1 *Aeromons caviae* were identified by the BD Phoenix automated identification and AST system. Almost all of the identified isolates were multidrug resistant (MDR) since we had selectively isolated the ESBL-producing *Enterobacteriaceae* from the fish samples with 1 µg/ml of cefotaxime in the MaConkey agar plate and selected the suspected colonies. Furthermore, we only selected MDR strains for further characterization by PCR for AGRs and then WGS. The *Klebsiella* isolates studied were susceptible to the majority of the antibiotics tested, while an intermediate MIC was observed for cephalosporins and fluoroquinolones; these isolates were classified as non-ESL strains. These non-ESBL strains are pathogenic bacteria and are devoid of extended-spectrum beta-lactamases (ESBLs). Compared to non-ESBL *K. pneumoniae*, ESBL-producing *K. pneumoniae* is more pathogenic and virulent. However, in most instances, this does not reduce the risk associated with *K. pneumoniae*. It was shown in one study that the potential risk factors linked to both forms of *Enterobacteriaceae* are quite comparable when comparing ESBL-producing and non-ESBL-producing *Enterobacteriaceae* in the clinical context. Only the treatment regimen differed in the usage of antibiotics for ESBL-producing and non-ESBL-producing patients (Bhavnani et al., 2006).

The presence of 30 different AMR genes, including genes associated with reduced permeability, antibiotic efflux, 16S rRNA methylases, and aminoglycoside-modifying enzymes, in the genomes studied, indicated potential hazards, especially when considering the abundance of plasmids that are known to carry AMR genes horizontally. The efflux pump systems, which include the *AcrAB* and *mdtK* systems, are also considered to be responsible for MDR in *K. pneumoniae* (Padilla et al., 2010; Meletis et al., 2012), and they belong to the resistance nodulation division (RND) class and multi-antimicrobial extrusion (MATE) family of efflux pumps, respectively (Wasfi et al., 2016). In all the genomes studied, the universal presence of these genes reflects the inherent transmissibility of the viruses. Unique cases of SHV variants have been reported in clinical settings globally (Liakopoulos et al., 2016). Similarly, in this particular study, different variants, such as *bla*_SHV-1_, *bla*_SHV-110,_ and *bla*_SHV-60_, were detected in the fish-sourced isolates, while *bla*_SHV-1,_ *bla*_SHV-106,_ *bla*_SHV-120,_ *bla*_SHV-134,_ *bla*_SHV-182_ and *bla*_SHV-187_ were detected in other reference genomes from food/environmental sources. The presence of different variants of *bla*_SHV_ genes in all the isolates is attributed to the organism’s ability to adapt to selective environmental pressure (Effah et al., 2020).

Several factors contribute to the virulence and pathogenicity of *K. pneumoniae*. Among these, the capsular serotype, lipopolysaccharide, iron-acquisition siderophores, and fimbrial and non-fimbrial adhesions are significant (Fuursted et al., 2012). Fimbrial adhesions play a major role in biofilm formation and are classified as virulence factors. The presence of type I and III fimbriae genes in the genome points to the ability of these isolates to adhere to host cells and form biofilms. The presence of the *fim* operon and *mrkA* gene in all the isolates contributed to the association of these genes with communities that induce pathogenicity (Alcántar-Curiel et al., 2013). The *mrkD* gene is a type 3 fimbrial adhesion gene that mediates agglutination and was found in all four genomes of fish origin. It is known to be rare in *K. pneumoniae* strains but highly conserved in *K. oxytoca* (Schurtz et al., 1994; Sebghati et al., 1998). This confirms the ability of the isolates to adhere to human renal/pulmonary epithelial cells or tissues, thus rendering them pathogenic. The absence of *rmpA* is a key feature of all the studied genomes, along with other reference genomes. Since *rmpA* is associated with virulence in *Klebsiella*, the absence of this particular virulence factor is intriguing and might be due to the draft nature of the genome. Of the present genomes from the food studied, 50% were O3b and the other 50% were O1v2. O1 is the most common serotype, followed by O3, while the O3b locus in particular is considered to be very rare according to studies of the global genome dataset (*n*=573) of *K. pneumoniae* (Follador et al., 2016).

Although our isolates did not show any carbapenemase or oxacillin resistance, the clonal type ST37 is indicative of its ability to transform into a highly epidemic clone. ST37 is a known high-risk international clone of *K. pneumoniae* with a greater ability to confer multidrug resistance to extended beta-lactamase, AmpC, or carbapenemase producers (Roe et al., 2019; Huynh et al., 2020). According to the definition by Huynh et al. (2020), “high-risk clones” are those that are represented at least 10 times in NCBI genomes, although reports of ST37 clones in the Indian context are very limited. There is a single report of an ST37 *K. pneumoniae* clinical isolate (either a urine or respiratory tract sample) harboring *bla*_NDM-1_ from Chennai Hospital, India (Giske et al., 2012). Archived data on sequence type 37 of *Klebsiella* spp. from fish as food origin is not available. The molecular characterization of NDM-1 *K. pneumoniae* isolates belonging to other STs has been reported from the neonatal ward of a tertiary care hospital in Agartala, Northeast India (Mukherjee et al., 2019). NDM-1-producing *K. pneumoniae* with ST37 clones were isolated from neonates at a Chinese children’s hospital (Zhu et al., 2016). The incidence of epidemic clone ST37 has been associated with nosocomial infections and has also been reported in canines in Japan (Taniguchi et al., 2017). A high prevalence of ESBL-associated ST37 clones of *K. pneumoniae* was reported in companion animals in China (Xia et al., 2017). Strains with high-risk clonal lineages can colonize people outside hospital settings (Holt et al., 2015). The present findings highlight the possibility of producing high-risk clones even from food sources, and to the best of our knowledge, this is the first Indian report on ST37 *K. pneumoniae* isolated from a non-human source, *i.e.*, fish. ST37 and ST192 clones of *K. pneumoniae* have been reported from the fecal samples of healthy individuals by Lepuschitz et al. (2020), who cited food as a possible source of these pathogens. Our study emphasizes the finding that food can act as a potential reservoir of high-risk clones, creating an ideal niche for the colonization of *K. pneumoniae* in the community. Guo et al. (2016) reported the presence of ST515 in *Klebsiella pneumoniae* isolated from ventilator-associated pneumonia patients in mainland China. Sequence type 515 was associated with non-hypermucoviscous strains of *K. pneumoniae* in this study. The IncR incompatibility group plasmid has been widely reported in clinical isolates worldwide. This particular plasmid is generally associated with MDR genes (Guo et al., 2016). In our case, the IncR plasmid was associated with ST515 clones along with the *bla*_SHV-1_ variant. The phylogenetic analysis indicated a distinct lineage for *K. pneumoniae* from fishes, although proximity was observed to one of the isolates and the reference genome Kp_HS11286. This study highlights the significance of the circulation of new STs in this fishery niche and its adaptation. However, the small number of samples (n=37) will not represent the complete picture of *K.pneumoniae* prevalence and the genetic diversity of its AMR. This preliminary study could shed light on the presence of AMR *K.pneumoniae* and more detailed studies with more AMR pathogens are warranted.

In summary, epidemiological risk factors such as the demography of a place, environment, food, livestock, and companion animals are known sources of *K. pneumoniae* infection. The present study revealed that food can be a potential reservoir of pathogenic *K. pneumoniae*. This is the first study to report various STs, namely, ST37, ST515, and ST192, and rare serotype O3b *K. pneumoniae* isolates from fish. Whole-genome sequencing is a technique that provides greater insights into the clonal complexity of pathogenic bacteria. However, a large number of samples should be used so as to obtain the true occurrences of MDR *K. pneumoniae* in fish samples. The elimination of virulent or antimicrobial-resistant strains of *Klebsiella* in food must be enforced at all stages of the food chain.

## Data Availability

All the relevant data such as raw data, samples, records and sequencing information (NGS) are available with the corresponding author and will be shared on request. Please address all correspondence concerning this article to gkshivraman@gmail.com.
