# Supplementary material for: Identification of serotype O3b and high-risk clone ST37 of Klebsiella pneumoniae revealed by comparative genomic analysis
Source: Front Cell Infect Microbiol. 2025 Jan 20;14:1517125. doi: 10.3389/fcimb.2024.1517125 (PMC11788149; doi:10.3389/fcimb.2024.1517125)
Supplement: Supplementary file 1 [file Table1.docx]

**Supplementary Table S1: ESBL producing *K.pneumoniae* and *E.coli* isolated from the retail market fish in Kochi, Kerala, India**

| **ESBL-producing isolates** | **Detail of Sample** | **Location** |
| --- | --- | --- |
| ***Klebsiella pneumoniae*** | | |
| CIFT-4 | *Tilapia etroplus* | Palluruthy Market, Kochi |
| CIFT-5 | Crab | Broadway Market, Kochi |
| CIFT-6 | Mackerel | Palluruthy Market, Kochi |
| CIFT-7 | Tilapia | Kalamasery retail market, Kochi |
| CIFT-8 | Etroplus | Munambam Fishing Harbour, Kochi |
| CIFT-10 | Clam | Kadvanthra Retail Market, Kochi |
| ***E. coli isolate ID*** | | |
| CIFT-1 | Sardine | Kadvanthra Retail Market, Kochi |
| CIFT-2 | Clam | Kadvanthra Retail Market, Kochi |
| CIFT-3 | Clam | Fort Kochi, Kochi |
| CIFT-4 | Mackerel | Valappu Market, Kochi |
| CIFT-5 | Crab | Munambam Fishing Harbour, Kochi |
| CIFT-6 | Catfish | Valappu Market, Kochi |
| CIFT-7 | Crab | Munambam Fishing Harbour, Kochi |
| CIFT-8 | Prawns | Kalamasery retail market, Kochi |
| CIFT-9 | Anchovy | Munambam Fishing Harbour, Kochi |
| CIFT-10 | Shrimp | Cheroy market, Kochi |
| CIFT-11 | Anchovy | Bismi Hyper Market, Kathirikadav, Kochi |
| CIFT-12 | Horse Mackerel | Valappu Market, Kochi |
| CIFT-13 | Clam | Broadway Market, Kochi |
| CIFT-14 | Prawns | Bismi Hyper Market, Kathirikadavu, Kochi |
| CIFT-15 | Prawns | Kalamasery Retail Market, Kochi |
